# Comparative Study of Fisetin-Loaded Poloxamer 407 and Poloxamer 188 Mixed Micelles as Nanocarrier Systems

**DOI:** 10.3390/molecules31101576

**Published:** 2026-05-09

**Authors:** Tomasz Przybylski, Joanna Czerniel, Aleksandra Majchrzak-Celińska, Barbara Jadach, Violetta Krajka-Kuźniak, Maciej Stawny

**Affiliations:** 1 Department of Pharmaceutical Chemistry, Poznan University of Medical Sciences, Rokietnicka 3, 60-806 Poznań, Poland; jczerniel@ump.edu.pl; 2 Doctoral School, Poznan University of Medical Sciences, Bukowska 70, 60-812 Poznań, Poland; 3 Department of Pharmaceutical Biochemistry, Poznan University of Medical Sciences, 3 Rokietnicka, 60-802 Poznań, Poland; majchrzakcelinska@ump.edu.pl (A.M.-C.); vkrajka@ump.edu.pl (V.K.-K.); 4 Department of Pharmaceutical Technology, Poznan University of Medical Sciences, 3 Rokietnicka, 60-802 Poznań, Poland; bjadach@ump.edu.pl

**Keywords:** fisetin, mixed polymeric micelles, Poloxamer 407, Poloxamer 188, nanocarrier-based drug delivery

## Abstract

Fisetin (FIS) is a bioactive flavonoid with antioxidant, anti-inflammatory, and anticancer activity, but its poor aqueous solubility and high lipophilicity limit its therapeutic use. In this study, three-component FIS-loaded mixed micelles based on Poloxamer 407 (P407) or Poloxamer 188 (P188), sodium deoxycholate, and Kolliphor HS15 or Kolliphor ELP were developed and comparatively evaluated. The formulations were prepared by the thin-film hydration method and characterized in terms of physicochemical properties, storage stability, solid-state properties, and in vitro biological activity. All freshly prepared formulations formed nanosized systems with high encapsulation efficiency. Although P188-based micelles showed smaller initial particle sizes, P407-based systems exhibited superior stability after lyophilization and rehydration. Formulations containing Kolliphor ELP showed the most favorable stability profile over 28 days of storage. FT-IR, TG, DSC, and XRPD analyses confirmed successful incorporation of FIS into the polymeric matrix and transformation of the drug into an amorphous or molecularly dispersed state. In vitro studies demonstrated that micellar encapsulation enhanced the cytotoxic activity of FIS against MICH-2 melanoma cells compared with the free compound, while P407-based systems showed a more favorable safety profile toward MRC-5 fibroblasts. These findings indicate that P407-based mixed micelles, particularly those containing Kolliphor ELP, may serve as promising nanocarriers for improving FIS delivery with potential relevance for dermal and anticancer applications.

## 1. Introduction

**Figure 1:**
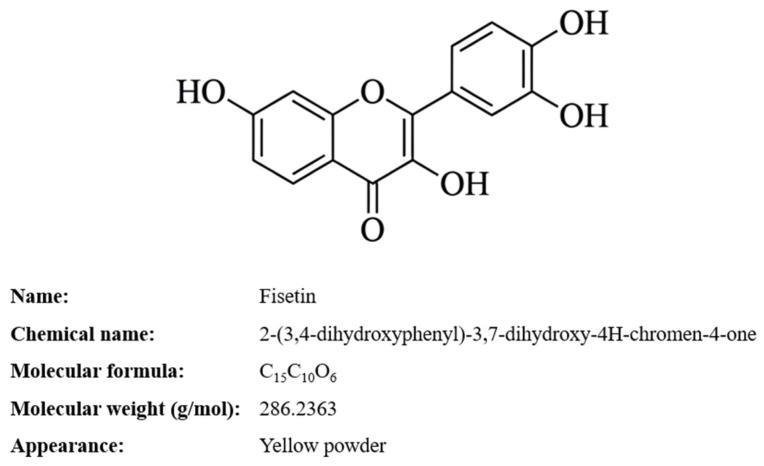
Chemical structure of fisetin.

Flavonols constitute an important subclass of naturally occurring polyphenolic compounds exhibiting a broad spectrum of biological activities [1]. Among them, fisetin (3,7,3,4-tetrahydroxyflavone, FIS), shown in Figure 1, has attracted considerable attention due to its antioxidant, anti-inflammatory, antiangiogenic, and anticancer properties [2]. FIS has demonstrated the ability to inhibit tumor growth, invasion, and migration; induce cell cycle arrest; modulate autophagy; and regulate key oncogenic signaling pathways, including MAPK, Ras, and NF-κB [2,3,4]. Furthermore, recent studies highlight its senotherapeutic potential, including both senolytic and senomorphic activity, particularly relevant in skin aging and tumor microenvironment modulation [5,6]. Despite its promising pharmacological profile, the clinical translation of FIS remains limited by its extremely poor aqueous solubility (~10 μg/mL), relatively high lipophilicity (log P ≈ 3.2), rapid metabolism to sulfates and glucuronides, and short systemic half-life following oral or intravenous administration. These properties result in low bioavailability and necessitate high therapeutic doses, potentially increasing the risk of adverse effects [1,2]. Therefore, the development of advanced drug delivery systems capable of improving FIS solubility, stability, and local bioavailability is essential [7].

Nanotechnology-based delivery systems have emerged as an effective strategy to overcome the limitations of poorly water-soluble flavonoids. FIS has been incorporated into various nanosystems, including liposomes [8], nanoemulsions [9], solid lipid nanoparticles [10], albumin nanoparticles [11], nanocochelates [12], cyclodextrin complexes [13], and polymeric micelles [14]. Among these platforms, polymeric mixed micelles (MMs) represent a particularly promising approach due to their ability to encapsulate hydrophobic compounds within a nanosized core–shell structure, improve aqueous solubility, and enhance drug stability while maintaining biocompatibility [14,15].

**Figure 2:**
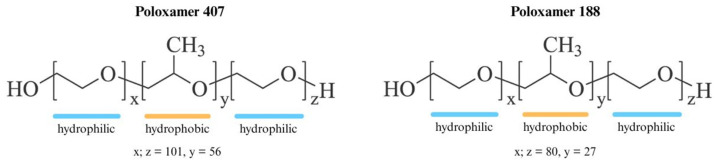
Chemical structures of Poloxamer 407 and Poloxamer 188.

Mixed micelles are formed by the self-assembly of at least two amphiphilic components in aqueous media. The incorporation of different surfactants or polymers enables modulation of micellar size, stability, surface properties, and drug loading capacity [15]. Block copolymers such as poloxamers (Pluronics^®^), including Poloxamer 407 (P407) and Poloxamer 188 (P188), composed of poly(ethylene oxide)–poly(propylene oxide)–poly(ethylene oxide) (PEO–PPO–PEO) triblock structures, are widely used as micellar carriers due to their excellent biocompatibility, low critical micelle concentration, and ability to form stable nanostructures. The chemical structures of P407 and P188 are presented in Figure 2. The hydrophobic PPO core enables encapsulation of lipophilic drugs, while the hydrophilic PEO corona provides steric stabilization and improves colloidal stability [16,17].

**Figure 3:**
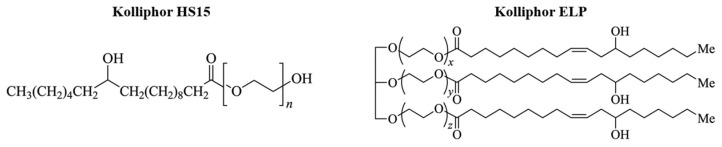
Chemical structures of Kolliphor HS15 and Kolliphor ELP.

The physicochemical properties of micelles can be further optimized by combining poloxamers with additional amphiphilic agents. Sodium deoxycholate, a bile salt with biosurfactant properties, can modify micellar polarity and reduce particle size, while PEG-based surfactants such as Kolliphor HS15 and Kolliphor ELP (Figure 3) enhance solubilization capacity and steric stabilization through their hydrophilic PEG chains. However, differences in molecular structure, degree of ethoxylation, and hydrophilic–lipophilic balance among these components may significantly influence micelle stability, aggregation behavior, and drug encapsulation efficiency [18,19,20].

Although numerous studies have described FIS-loaded nanosystems [8,9,10,11,12,13,14], comparative data evaluating the impact of different poloxamer types within three-component mixed micelles remain limited. The available reports focus almost exclusively on the use of P407, while the role of other poloxamers, such as P188, in formulations containing FIS remains poorly understood [21,22,23]. In particular, systematic comparison between both Poloxamers in combination with sodium deoxycholate and distinct Kolliphor derivatives has not been thoroughly investigated in the context of FIS delivery. Accordingly, this study aimed to develop an alternative nanoformulation of FIS using the aforementioned poloxamers, while modifying the amounts of the ingredients and types of surfactants. Given the growing interest in nanocarrier-based strategies for melanoma management and dermal applications, the development of stable, nanosized FIS formulations with improved physicochemical properties may represent a valuable therapeutic approach. Therefore, the aim of the present study was to design and comparatively evaluate innovative three-component mixed micellar systems based on P407 or P188, sodium deoxycholate, and either Kolliphor HS15 or Kolliphor ELP as nanocarriers for FIS. The formulations were characterized in terms of mean particle size (MPS), polydispersity index (PDI), zeta potential (ZP), encapsulation efficiency (EE), and drug loading (DL). Selected systems were further analyzed using FT-IR spectroscopy, thermogravimetric analysis (TG), differential scanning calorimetry (DSC), and X-ray powder diffraction (XRPD) to assess molecular interactions and physical state transformation. Additionally, the biological activity and safety profile of FIS and FIS-loaded micelles were evaluated in melanoma and non-cancerous cell models.

## 2. Results

### 2.1. Physicochemical Properties of FIS-Loaded Mixed Micelles

Freshly prepared FIS-loaded mixed micelles (FMs) were evaluated prior to lyophilization to determine their physicochemical characteristics. Each formulation contained a fixed amount of API (8 mg) and sodium deoxycholate (160 mg). The formulations were grouped according to the main block copolymer used: P407 (FM1–FM4) and P188 (FM5–FM8). The ratio of Poloxamers to Kolliphores in formulations FM1, FM2, FM5, and FM6 was 1:1, whereas the other formulations contained 160 mg of polymers and 112 mg of surfactants. The detailed physicochemical parameters are presented in Table 1. All formulations formed nanosized particles with relatively narrow size distributions. Particle size values were within the nanometric range for all systems, while P188-based formulations generally produced smaller particles compared to P407-based systems. Encapsulation efficiency remained high across all formulations, with the highest value observed for FM6. All systems exhibited negative zeta potential values, indicating the presence of negatively charged surface components (Table 1).

### 2.2. Stability of Rehydrated Lyophilized Micelles

**Figure 4:**
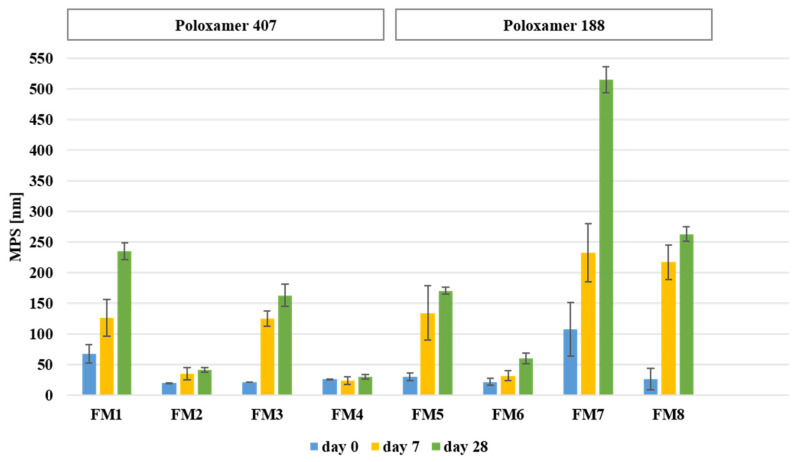
Changes in mean particle size (MPS) of rehydrated FIS-loaded mixed micelles during 28 days of storage at 4 ± 1 °C.

**Figure 5:**
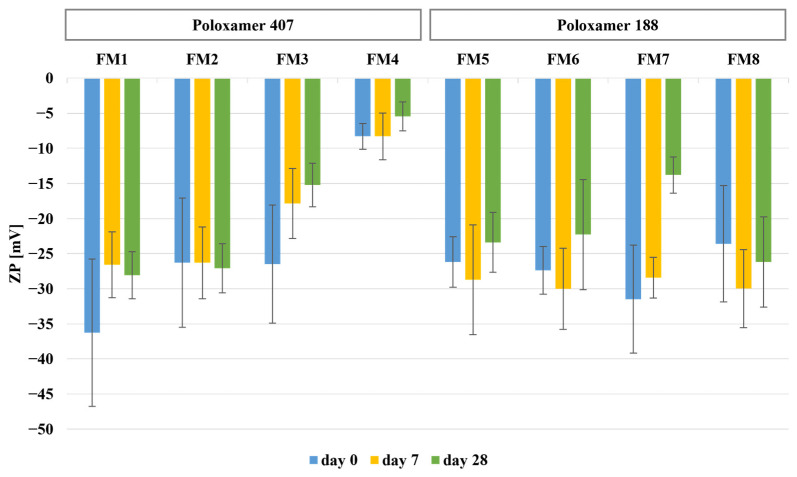
Changes in zeta potential (ZP) of rehydrated FIS-loaded mixed micelles during 28 days of storage at 4 ± 1 °C.

Storage stability of the developed formulations was evaluated after rehydration at predefined time points (0, 7, and 28 days). The physicochemical stability data are summarized in Table 2 and Table 3 and Figure 4 and Figure 5. Immediately after rehydration, all formulations formed nanoscale dispersions with particle sizes comparable to those observed prior to lyophilization (Table 2).

Immediately after rehydration (Table 2), the smallest mean particle sizes were observed for FM2, FM3, and FM6 (approximately 20–22 nm), while slightly larger particles were noted for FM4, FM5, and FM8 (approximately 26–30 nm). In contrast, FM7 showed a markedly higher particle size (107.98 ± 43.48 nm). PDI values ranged from 0.22 to 0.41, indicating acceptable size distribution for polymeric micelles. All formulations exhibited negative zeta potential values (−8.3 to −36.3 mV), suggesting acceptable colloidal stability after rehydration. Encapsulation efficiency remained high for all formulations (approximately 65–75%) and was consistent with the stability data obtained during storage (Table 3).

During the 28-day storage period, formulation-dependent changes in particle size were observed. Formulations containing Kolliphor ELP (FM2, FM4, FM6) maintained particle sizes within the nanoscale range, whereas selected P188-based formulations, particularly FM7 and FM8, showed more pronounced size increases (Figure 4). PDI values remained within the typical range for polymeric micelles, with only slight increases observed after storage (Table 3). All formulations maintained negative zeta potential values throughout the study period (Figure 5), with relatively stable values observed for FM2, FM4, and FM6, while greater fluctuations were noted for FM7 and FM8. Encapsulation efficiency remained high and stable throughout the study period, with values remaining above 62% after 28 days (Table 3). Similarly, drug loading remained within a narrow range (approximately 1.33–1.59%) across all formulations and time points.

### 2.3. Solid-State Characterization of Lyophilized FIS-Loaded Mixed Micelles

**Figure 6:**
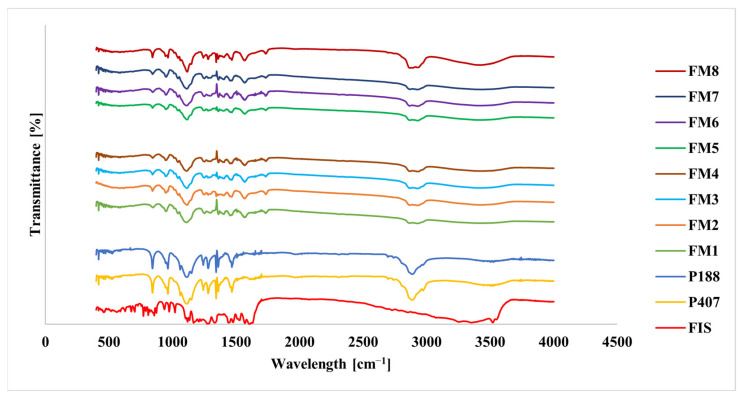
FT-IR spectra of FIS, poloxamers (P407 and P188), and lyophilized FIS-loaded mixed micelles.

**Figure 7:**
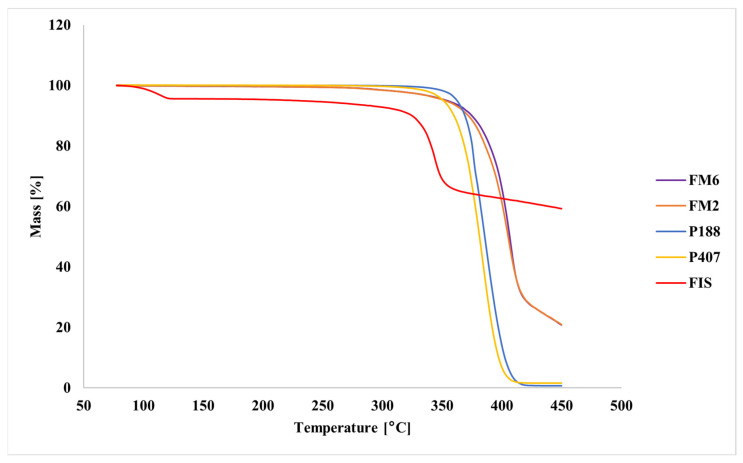
TG thermograms of FIS, poloxamers (P407 and P188), and lyophilized FIS-loaded mixed micelles.

**Figure 8:**
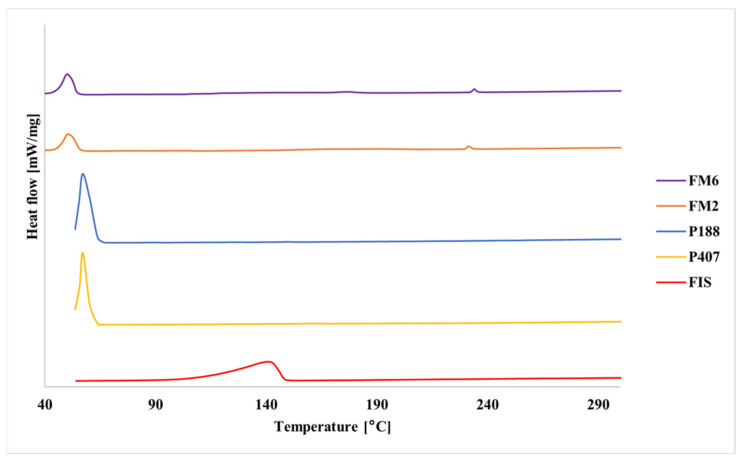
DSC curves of FIS, poloxamers (P407 and P188), and lyophilized FIS-loaded mixed micelles.

**Figure 9:**
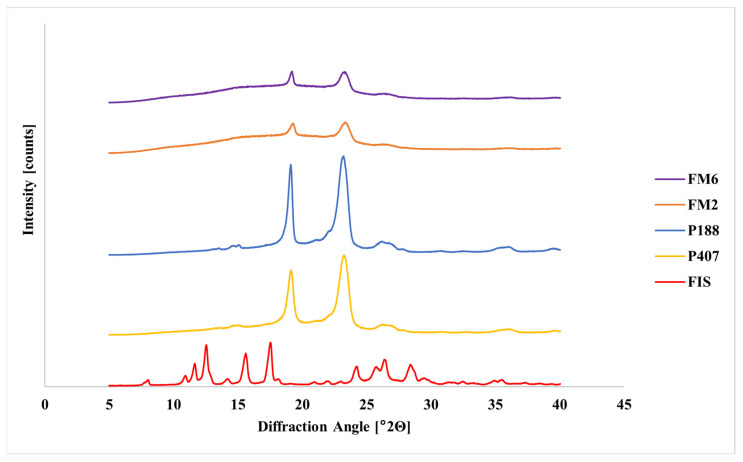
XRPD diffractograms of FIS, poloxamers (P407 and P188), and lyophilized FIS-loaded mixed micelles.

To further investigate the structural properties of the lyophilized systems were analyzed using FT-IR, TG, DSC, and XRPD. Representative spectra and thermograms are shown in Figure 6, Figure 7, Figure 8 and Figure 9. FT-IR spectra of pure FIS showed characteristic absorption bands corresponding to hydroxyl groups in the range of 3200–3500 cm^−1^, a strong carbonyl stretching band at approximately 1610–1620 cm^−1^, and aromatic C=C vibrations in the 1500–1600 cm^−1^ region (Figure 6, Table 4). The spectra of both poloxamers were dominated by a strong band at approximately 1105 cm^−1^ corresponding to C-O-C stretching vibrations, with additional bands observed at 2880–2920 cm^−1^ and 1340–1470 cm^−1^. The spectra of the lyophilized formulations were dominated by characteristic polymer bands, while signals corresponding to FIS were reduced in intensity or partially masked (Figure 6).

Thermogravimetric analysis showed that pure FIS exhibited high thermal stability, with the main mass loss occurring above approximately 340 °C (Figure 7). In contrast, pure poloxamers showed rapid decomposition within the temperature range of 350–400 °C. The lyophilized formulations remained thermally stable up to approximately 250 °C, followed by progressive mass loss at higher temperatures.

DSC curves of pure poloxamers showed sharp endothermic peaks in the range of 50–60 °C corresponding to melting of crystalline PEO domains (Figure 8). Pure FIS showed a broad endothermic transition above 100 °C. In the lyophilized formulations, the melting transitions of poloxamers were shifted and reduced in intensity.

XRPD analysis of pure FIS revealed numerous sharp diffraction peaks in the 2θ range of 10–30°, confirming its crystalline structure (Figure 9). Pure poloxamers showed characteristic reflections near 19° and 23°. In contrast, diffraction peaks characteristic of FIS were not observed in the lyophilized formulations, while polymer reflections remained detectable.

### 2.4. In Vitro Cytotoxicity Assay

**Figure 10:**
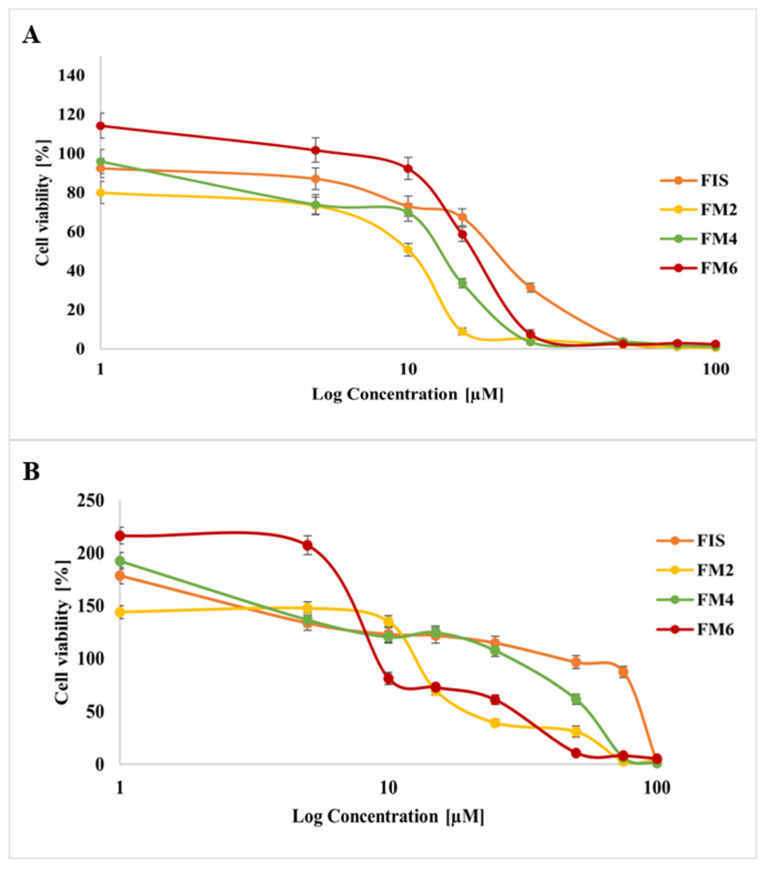
Cytotoxic activity of free FIS and FIS-loaded mixed micelles (FM2, FM4, FM6) in (**A**) MICH-2 melanoma cells and (**B**) MRC-5 fibroblasts after 48 h incubation.

Based on physicochemical characterization and stability studies, three representative formulations (FM2, FM4, and FM6) were selected for biological evaluation. These systems demonstrated the most favorable balance between nanoscale particle size, encapsulation efficiency, and stability after lyophilization and storage. Importantly, all selected systems contained the same surfactant, which allowed for a more controlled comparison of formulation performance, while at the same time representing distinct profiles in terms of key physicochemical characteristics, thereby enabling a comparative assessment of how these differences may influence biological activity. In contrast, other formulations, although initially comparable in size, exhibited a significant increase in particle size over time, indicating reduced stability and making them less suitable for further biological evaluation. Therefore, FM2, FM4, and FM6 were considered the most appropriate candidates, as they ensured both high drug loading capacity and sufficient physicochemical stability, which are critical factors for reliable assessment of biological activity. The cytotoxic activity of selected FIS-loaded micelles (FM2, FM4, FM6) and free FIS was evaluated in MICH-2 melanoma cells and MRC-5 fibroblasts. The results are presented in Figure 10. All tested samples showed concentration-dependent effects on cell viability. In MICH-2 melanoma cells, free FIS produced a gradual decrease in cell viability with increasing concentration. In contrast, micellar formulations demonstrated enhanced cytotoxic activity, resulting in a more pronounced reduction in viability at lower concentrations. Among the tested formulations, FM2 showed the strongest cytotoxic effect, followed by FM6, while FM4 demonstrated a more moderate decrease in melanoma cell viability (Figure 10A). In MRC-5 fibroblasts, higher cell viability was generally observed at lower and intermediate concentrations compared to melanoma cells. Free FIS maintained relatively high fibroblast viability over a broad concentration range. Among the micellar systems, FM4 showed the highest fibroblast viability, whereas FM6 demonstrated the strongest reduction in viability at higher concentrations (Figure 10B). Overall, the results indicate formulation-dependent differences in cytotoxic response between cancerous and non-cancerous cells.

Reference (blank) micelles without FIS also exhibited concentration-dependent effects on cell viability (Figure S1). In MICH-2 melanoma cells, all blank formulations caused a marked decrease in viability at higher concentrations, with near-complete loss of viability above ~15–20 µM. In MRC-5 fibroblasts, a similar trend was observed, although viability was generally lower already at baseline and further decreased with increasing concentration. Overall, these results indicate that the micellar carriers themselves contribute to cytotoxic effects, particularly at higher concentrations.

## 3. Discussion

The present study demonstrates that the type of poloxamer plays a critical role in determining the physicochemical behavior, storage stability, and biological performance of FIS-loaded mixed micelles. Although both P407 and P188 enabled the formation of nanosized systems with high encapsulation efficiency, clear differences emerged after lyophilization and rehydration, indicating that polymer molecular architecture strongly influences nanocarrier performance. All freshly prepared formulations formed nanosized systems with narrow size distributions and high encapsulation efficiency, confirming that the thin-film hydration method enabled efficient incorporation of hydrophobic FIS. Similar observations have been reported for other FIS nanocarriers, including liposomes, nanoemulsions, and polymeric micelles, where nanoscale size and efficient drug incorporation improved the pharmaceutical performance of poorly soluble flavonoids [8,9,14].

P188-based systems generally produced smaller particles than P407-based formulations, likely due to their lower molecular weight and shorter PPO block, which may favor the formation of more compact micellar cores. However, this smaller size did not translate into superior storage stability. After lyophilization and rehydration, the most stable systems were those containing Kolliphor ELP, whereas HS15-containing formulations showed greater aggregation. This suggests that long-term stability depends not only on polymer type but also on surfactant structure. The improved stability of Kolliphor ELP systems may be attributed to their higher degree of ethoxylation and longer PEG chains, which form a thicker hydrated corona and provide more effective steric stabilization. Similar stabilization mechanisms have been reported for PEG-containing surfactants, which reduce interparticle interactions and improve colloidal robustness [24,25,26,27]. In contrast, P188-based systems appeared more susceptible to structural changes during freeze-drying, suggesting that the shorter PPO block may result a less robust micellar core under processing stress [28]. Zeta potential results further support this interpretation. Although all systems exhibited negative surface charge, the most stable formulations maintained relatively consistent ZP values during storage. However, in PEG-containing systems steric stabilization plays a major role, and moderate ZP values do not necessarily indicate poor stability due to shielding effects of hydrated polymer chains [29,30].

The high EE observed before and after lyophilization is consistent with the affinity of FIS for the hydrophobic domains of mixed micelles. Stable EE and DL values during storage indicate preserved drug retention despite structural stress associated with drying and rehydration. Such stability is essential for maintaining reproducible dosing and formulation shelf-life [24,31]. Comparable EE values have been reported for other mixed micellar systems containing hydrophobic drugs, including P188/sodium deoxycholate micelles described by Palei et al. [32].

Solid-state analyses further confirmed successful incorporation of FIS into the polymeric matrix. FT-IR results indicated good compatibility between formulation components and suggested intermolecular interactions, most likely hydrogen bonding between FIS and poloxamer chains. Thermal and XRPD analyses consistently indicated loss of crystalline FIS structure and formation of an amorphous or molecularly dispersed drug state. Such transformations are commonly associated with improved apparent solubility and dissolution behavior of poorly soluble drugs [33,34,35,36,37,38,39,40,41,42,43,44].

Importantly, the physicochemical differences between formulations were reflected in their biological performance. Micellar formulations enhanced the cytotoxic activity of FIS in melanoma cells compared with the free compound, which may result from improved solubilization and cellular availability. Similar improvements in anticancer activity following nanoencapsulation of hydrophobic compounds have been widely reported [7,45,46,47].

Differences between formulations also suggest that nanocarrier architecture may influence therapeutic selectivity. P407-based systems combined strong anticancer activity with relatively favorable safety toward fibroblasts, whereas P188-based systems showed higher toxicity toward normal cells. However, in the absence of experimental data on drug release kinetics, these interpretations remain indirect. It can be hypothesized that the larger hydrophobic core and improved structural stability of P407 micelles may be associated with more controlled drug release, whereas P188 systems may potentially favor faster drug availability and less selective effects; however, these assumptions require further experimental verification. These findings are in line with previous reports showing more pronounced cytotoxicity of FIS toward melanoma cells with limited toxicity toward normal fibroblasts [42].

Taken together, the results indicate that the performance of FIS-loaded mixed micelles depends on a balance between core structure, steric stabilization, drug retention, and resistance to processing stress. P407-based systems, particularly those containing Kolliphor ELP, provided the most advantageous overall profile, combining good stability, preserved drug loading, and favorable biological activity. These findings highlight the importance of polymer selection as a key parameter in the design of mixed micellar nanocarriers.

This study has several limitations. A principal limitation of the present study is the absence of experimental evaluation of FIS release kinetics; consequently, the relationship between micellar structure and intracellular drug availability, as well as the interpretation of observed differences in biological response, remains indirect and should be regarded as tentative. Furthermore, the biological assessment remains preliminary, as it was restricted to three selected formulations and in vitro experiments conducted using a single melanoma cell line and one non-cancerous fibroblast model. In addition, the biological evaluation was limited to two-dimensional cell culture systems, and stability studies were confined to a 28-day period. Therefore, any conclusions regarding therapeutic selectivity and dermal applicability should be interpreted with caution and require further validation in more advanced biological systems. Future investigations should include comprehensive release kinetics, mechanistic cellular studies, extended stability evaluation, and validation in more advanced biological models. Overall, this study demonstrates that although P188 favored smaller initial particle size, P407-based formulations provided a more favorable balance between physicochemical stability and biological performance after lyophilization and rehydration. In particular, systems containing Kolliphor ELP emerged as the most robust carriers. These findings provide guidance for the rational design of micellar nanocarriers for FIS and other poorly soluble flavonoids intended for anticancer and dermal applications.

The particle size and colloidal characteristics observed in this study may be considered beneficial for topical application, as nanoscale systems—particularly within the range below ~100–250 nm—have been reported to enhance interaction with the skin barrier and promote deposition not only within the stratum corneum but also in deeper skin layers. Furthermore, appropriate colloidal stability and surface properties are known to support homogeneous distribution and prolonged residence time on the skin surface [48,49].

In the context of melanoma-targeted delivery, such physicochemical properties are particularly relevant, as effective delivery systems should enable penetration into viable epidermis and upper dermis, where melanocytes reside and where melanoma progression involves interactions with dermal components, including fibroblasts. Nanoparticles with suitable size, high surface-to-volume ratio, and tunable surface characteristics may enhance cellular uptake and improve drug accumulation within tumor cells while potentially limiting effects on surrounding healthy cells [50,51]. In this context, the observed cytotoxic activity toward melanoma cells, together with the response of fibroblasts, provides preliminary evidence supporting the biological relevance of the developed system.

Nevertheless, it should be emphasized that in vitro results obtained on melanoma and fibroblast cell lines do not directly confirm effective dermal penetration or in vivo tumor targeting. Therefore, further dedicated studies—particularly involving skin permeation models and in vivo melanoma systems—are required to verify whether the nanoparticles can reach and accumulate in relevant skin compartments and exert therapeutic effects under physiological conditions [52,53].

## 4. Materials and Methods

### 4.1. Materials

FIS was purchased from AmBeed (Buffalo Grove, IL, USA), while P407, P188, sodium deoxycholate, Kolliphors HS15, and Kolliphor ELP were purchased from Sigma-Aldrich (Taufkirchen, Germany). Anhydrous ethanol, methanol, and acetonitrile were purchased from POCH S.A. (Gliwice, Poland). All chemicals were analytical or high-performance liquid chromatographic grade. Non-cancerous MRC-5 were obtained from ATCC (Manassas, VA, USA). The MICH-2 cell line was obtained from Poznan University of Medical Science, Department of Medical Biotechnology.

### 4.2. Preparation and Physicochemical Characterization of Freshly Prepared FIS-Loaded Mixed Micelles

Eight FIS-loaded mixed micellar formulations with a nominal FIS concentration of 1 mg/mL were prepared using the thin-film hydration method. Appropriate amounts of components (Table 5) were weighed into round-bottom flasks and dissolved in 8 mL of anhydrous ethanol using an ultrasonic bath at 37 °C. The organic solvent was removed using a rotary evaporator at 42 °C for 24 min until a thin polymeric film was formed. The flasks were then kept overnight at room temperature to ensure complete removal of residual solvent. The dried films were subsequently hydrated with 8 mL of distilled water and stabilized in a shaking water bath at 37 °C for 60 min to allow spontaneous micelle formation.

The freshly prepared FIS-loaded mixed micelles were characterized in terms of mean particle size (MPS), polydispersity index (PDI), zeta potential (ZP), and encapsulation efficiency (EE). Mean particle size and PDI were determined using dynamic light scattering (DLS) with a Zetasizer Nano ZS (Malvern Instruments Ltd., Worcester, UK) equipped with a 633 nm laser operating at a fixed scattering angle of 173°. Measurements were performed at 25 °C. Zeta potential was determined using electrophoretic light scattering with the same instrument. For measurements, 100 μL of micellar dispersion was diluted to 10 mL with distilled water and transferred into polycarbonate cuvettes. All measurements were performed in triplicate and reported as mean ± standard deviation. Encapsulation efficiency (EE) was determined immediately after rehydration and during storage according to the procedure described in Section 4.8.

### 4.3. Lyophilization, Rehydration and Storage Stability of FIS-Loaded Mixed Micelles

Freshly prepared micellar dispersions were subjected to freeze-drying to evaluate the effect of lyophilization on their physicochemical stability. The obtained lyophilizates were stored at 4 ± 1 °C and analyzed at predefined time points (0, 7, and 28 days). Prior to analysis, lyophilized samples were rehydrated with distilled water to the original volume and gently mixed until complete redispersion was achieved. The physicochemical properties of rehydrated micelles were determined using the same analytical procedures as described for freshly prepared formulations (Section 4.2). Encapsulation efficiency (EE) and drug loading (DL) were determined immediately after rehydration and during storage according to the procedure described in Section 4.8.

### 4.4. Fourier Transform Infrared (FT-IR) Spectroscopy

Infrared spectra in the range from 4000 to 400 cm^−1^ were plotted using an IRAffinity-IS Fourier transform infrared spectrophotometer (Shimadzu, Kyoto, Japan). 1.0 mg of the tested substances and lyophilizates and 300.0 mg of potassium bromide were weighed on an analytical balance. The mixtures were then transferred to a matrix, which was placed in a hydraulic press and tableted under a pressure of 8 atmospheres for 9 min.

### 4.5. Thermogravimetric Analysis (TG)

The thermal stability of FIS and lyophilizates was determined using a TG 209 F3 Tarsus^®^ micro-thermobalance (Netzsch, Selb, Germany). Approximately 6–8 mg of each sample was weighed into crucibles on an analytical balance and tested. Measurements were carried out in a nitrogen atmosphere. The heating rate of the samples was a constant value of 10.0 °C/min over the temperature range of 50–450 °C. Proteus 8.0 software (Netzsch, Selb, Germany) was used to analyze the obtained results.

### 4.6. Differential Scanning Calorimetry (DSC)

Calorimetric analysis was performed using a model DSC 214 Polyma (Netzsch, Selb, Germany) in a nitrogen atmosphere. Approximately 5–8 mg of each sample was weighed into hermetically sealed crucibles with an opening and heated at a rate of 10 °C/min. The DSC thermograms were analyzed using Proteus 8.0 software (Netzsch, Selb, Germany).

### 4.7. X-Ray Powder Diffraction (XRPD)

Powder X-ray diffraction using a Bruker D2 Phaser diffractometer (Bruker, Berlin, Germany) was used to verify the physical state of the following samples. Pure substances (FIS, P407, P188) and the obtained formulations were analyzed. The diffractograms were recorded over the range 5° to 40° 2Θ with a step of 0.02° 2Θ and a counting rate of 2 s·step^−1^.

### 4.8. FIS Quantitative Analysis

The FIS content in mixed micelles was determined using HPLC according to a modified method described by Sobczak et al. [44]. To determine drug content, approximately 8 mg of FIS-loaded micelles were dissolved in methanol and diluted to 5 mL to disrupt micellar structures and release the encapsulated drug. The obtained solution was filtered and analyzed. Chromatographic analysis was performed using an Agilent 1260 Infinity II system (Agilent Technologies, Santa Clara, CA, USA) equipped with a four-component pump, a linear degasser, a vial sampler (maintained at 25 ± 0.8 °C), a column oven (35 ± 0.8 °C), and a diode array detector (DAD) set at 360 nm. Separation was achieved on a C18 column (Luna C18(2), 150 × 4.6 mm, 5 μm; Phenomenex, Torrance, CA, USA). Gradient elution was performed using 0.1% phosphoric acid (A) and acetonitrile (B) at a flow rate of 1.0 mL/min. The gradient program was as follows: 0–11 min: 25 → 70% B, 11 → 12 min: 70–25% B, 12 → 15 min: 25% B. FIS concentration was determined using a calibration curve in the range 0.01–0.1 mg/mL.

The EE and DL of FIS were determined by direct measurements of its concentration in nanocarriers. The EE and DL of FIS in carriers were calculated using Equations (1) and (2):

(1) $$EE=\;\frac{Mass\;of\;encapsulated\;drug}{Total\;mass\;of\;drug\;added}\times 100$$

(2) $$DL=\frac{Mass\;of\;encapsulated\;drug}{Total\;mass\;of\;formulation}\times 100$$

### 4.9. Cell Culture Experiments

Human melanoma MICH-2 cell line and non-cancerous human fibroblast MRC-5 cell line were cultured in Dulbecco’s Modified Eagle’s Medium (DMEM, Sigma-Aldrich, St. Louis, MO, USA) containing 10% fetal bovine serum (EURx, Gdansk, Poland) and 1% antibiotic solution (Sigma-Aldrich, St. Louis, MO, USA) at 37 °C, in a humidified 5% CO_2_ atmosphere.

Cell viability was evaluated using the MTT assay. Cells were seeded in 96-well plates at a density of 10,000 cells per well and incubated for 48 h. Subsequently, free FIS and selected FIS-loaded micelles (FM2, FM4, FM6) at various concentrations were added and incubated for 48 h. After incubation, cells were washed with PBS and incubated for 4 h with medium containing 0.5 mg/mL MTT reagent. Formazan crystals were dissolved in acidic isopropanol and absorbance was measured at 570 nm with background correction at 690 nm. Each experiment was performed in triplicate.

## 5. Conclusions

The present study demonstrates that the type of poloxamer significantly influences the physicochemical properties, stability, and biological performance of FIS-loaded mixed micelles. Importantly, the results highlight a practical trade-off between particle size and post-processing stability, which should be considered in the design of such systems. Although P188-based systems produced smaller initial particle sizes, which may be advantageous for increasing surface area and potentially enhancing bioavailability, P407-based formulations exhibited superior stability after lyophilization and rehydration, making them more suitable for applications requiring improved robustness during processing and storage. In particular, micelles containing Kolliphor ELP showed the most favorable balance between nanoscale size, drug retention, and biological activity. Furthermore, nanoencapsulation was associated with increased cytotoxic activity of FIS against melanoma cells compared to the free compound, while maintaining an acceptable safety profile toward normal fibroblasts under the applied experimental conditions. These findings suggest that the rational selection of polymer composition may represent an important factor in the optimization of mixed micellar drug delivery systems and should be considered in the context of the intended application, particularly with respect to the relative importance of particle size and stability. Overall, the developed P407-based mixed micelles represent promising nanocarriers for improving the delivery of poorly soluble flavonoids such as FIS, particularly in formulations where stability is a critical factor. These findings may support the development of FIS formulations for topical melanoma therapy.

## Figures and Tables

**Table 1:** Physicochemical properties of freshly prepared FIS-loaded mixed micelles.

Formulation Type	Sample	MPS ± SD [nm]	PDI ± SD [−]	ZP ± SD [mV]	EE ± SD [%]
P407-based mixed micelles	FM1	49.72 ± 8.45	0.30 ± 0.07	−35.0 ± 7.1	67.97 ± 3.05
	FM2	19.70 ± 0.79	0.33 ± 0.01	−29.1 ± 5.9	84.02 ± 2.77
	FM3	22.15 ± 1.11	0.35 ± 0.02	−18.6 ± 9.5	75.16 ± 6.00
	FM4	33.45 ± 13.47	0.29 ± 0.02	−11.6 ± 2.5	77.51 ± 2.58
P188-based mixed micelles	FM5	23.18 ± 9.90	0.29 ± 0.07	−33.3 ± 13.4	74.36 ± 3.39
	FM6	14.60 ± 1.05	0.34 ± 0.04	−32.1 ± 7.5	87.14 ± 2.92
	FM7	16.77 ± 0.36	0.31 ± 0.01	−18.2 ± 3.4	80.93 ± 1.62
	FM8	37.19 ± 18.96	0.23 ± 0.09	−27.1 ± 17.6	80.35 ± 3.15

**Table 2:** Physicochemical properties of rehydrated FIS-loaded mixed micelles.

Formulation Type	Sample	MPS ± SD [nm]	PDI ± SD [−]	ZP ± SD [mV]	EE ± SD [%]
P407-based mixed micelles	FM1	68.19 ± 15.18	0.39 ± 0.25	−36.3 ± 10.5	65.15 ± 0.95
	FM2	19.73 ± 0.27	0.35 ± 0.01	−26.3 ± 9.2	74.18 ± 1.30
	FM3	21.61 ± 0.54	0.41 ± 0.01	−26.5 ± 8.4	64.86 ± 0.45
	FM4	26.28 ± 0.76	0.36 ± 0.06	−8.3 ± 1.8	72.03 ± 0.34
P188-based mixed micelles	FM5	30.20 ± 6.24	0.41 ± 0.17	−26.2 ± 3.6	64.78 ± 0.53
	FM6	22.00 ± 5.64	0.32 ± 0.09	−27.4 ± 3.4	74.78 ± 0.47
	FM7	107.98 ± 43.48	0.22 ± 0.08	−31.5 ± 7.7	68.88 ± 0.31
	FM8	29.46 ± 17.67	0.37 ± 0.16	−23.6 ± 8.3	73.46 ± 0.38

**Table 3:** Encapsulation efficiency (EE) and drug loading (DL) of rehydrated FIS-loaded mixed micelles during 28 days of storage at 4 ± 1 °C.

Formulation Type	Sample	Day 0		Day 7		Day 28	
		EE [%]	DL [%]	EE [%]	DL [%]	EE [%]	DL [%]
P407-based mixed micelles	FM1	65.15 ± 0.95	1.38 ± 0.02	64.49 ± 2.49	1.37 ± 0.05	62.71 ± 0.61	1.33 ± 0.01
	FM2	74.18 ± 1.30	1.55 ± 0.03	74.92 ± 0.71	1.57 ± 0.01	74.80 ± 0.24	1.57 ± 0.01
	FM3	64.86 ± 0.45	1.37 ± 0.01	64.84 ± 0.68	1.37 ± 0.01	64.75 ± 0.38	1.37 ± 0.01
	FM4	72.03 ± 0.34	1.54 ± 0.02	72.64 ± 0.46	1.56 ± 0.01	72.97 ± 0.66	1.56 ± 0.01
P188-based mixed micelles	FM5	64.78 ± 0.53	1.41 ± 0.01	64.52 ± 0.43	1.40 ± 0.01	65.09 ± 1.02	1.41 ± 0.02
	FM6	74.79 ± 0.47	1.58 ± 0.01	74.32 ± 1.02	1.57 ± 0.02	75.27 ± 0.52	1.59 ± 0.01
	FM7	68.88 ± 0.31	1.48 ± 0.01	69.06 ± 0.33	1.48 ± 0.01	68.77 ± 0.45	1.47 ± 0.01
	FM8	73.46 ± 0.38	1.59 ± 0.01	73.08 ± 0.97	1.59 ± 0.02	73.56 ± 1.25	1.57 ± 0.01

**Table 4:** Characteristic FT-IR absorption bands of FIS and poloxamer-based formulations, including corresponding wavenumbers, types of molecular vibrations, and functional group assignments.

Sample	Wavenumber [cm^−1^]	Types of Molecular Vibrations	Functional Group Assignment
FIS	3200–3500 (wide)	Stretching	Hydroxyl groups -OH (phenolic)
	1618	Stretching C=O	The carbonyl group in the flavonoid ring
	1512; 1565; 1604	Stretching C=C	Aromatic rings
	1278	Stretching C-O	Phenolic bonds
P407/P188	2884	Stretching C-H	Aliphatic groups CH_2_. CH_3_
	1466	Bending C-H	Methyl/methylene groups (scissoring)
	1342	Bending C-H	CH_2_ groups (wagging/twisting)
	1103	Stretching C-O-C	Ether bonds (polymer backbone)
	842 962	Rocking	Chain backbone PEO/PPO
FIS-loaded mixed micelles	3300–3500	Stretching -OH	A shift in the O-H stretching band
	2886	Stretching C-H	Polymer signal dominance
	1610–1620	Stretching C=O	Weakened/shifted FIS signal
	1105	Stretching C-O-C	Main spike of the polymer matrix
Kolliphors HS15/ELP	3400–3500 (wide)	Stretching O-H	Hydroxyl groups (PEG, fatty acid residues)
	2920; 2850	Stretching C-H	Aliphatic CH_2_/CH_3_ groups
	1730–1745	Stretching C=O	Ester groups
	1465	Bending C-H	CH_2_ scissoring
	1375	Bending C-H	CH_3_ symmetric deformation
Kolliphor ELP	~1650	Stretching C=C	Unsaturated fatty acids (ricinoleic acid)
	1240–1170	Stretching C-O	Ester bonds (more complex structure)

**Table 5:** Composition of FIS-loaded micelles.

Sample	Fisetin	Sodium Deoxycholate	Poloxamer 407	Poloxamer 188	Kolliphor HS15	Kolliphor ELP
	[mg]					
FM1	8.0	160.0	136.0	-	136.0	-
FM2	8.0	160.0	136.0	-	-	136.0
FM3	8.0	160.0	160.0	-	112.0	-
FM4	8.0	160.0	160.0	-	-	112.0
FM5	8.0	160.0	-	136.0	136.0	-
FM6	8.0	160.0	-	136.0	-	136.0
FM7	8.0	160.0	-	160.0	112.0	-
FM8	8.0	160.0	-	160.0	-	112.0

## Data Availability

The raw data supporting the conclusions of this article will be made available by the authors on request.

## Supplementary Materials

The following supporting information can be downloaded at: https://www.mdpi.com/article/10.3390/molecules31101576/s1, Figure S1: Cytotoxic activity of mixed micelles without FIS (blank FM2, FM4, and FM6) in (A) MICH-2 melanoma cells and (B) MRC-5 fibroblasts after 48 h incubation.

## Abbreviations

The following abbreviations are used in this manuscript:

DLS	Dynamic Light Scattering
DL	Drug Loading
DSC	Differential Scanning Calorimetry
EE	Encapsulation Efficiency
FIS	Fisetin
FMs	FIS-loaded Mixed Micelles
FT-IR	Fourier Transform Infrared Spectroscopy
HPLC	High-Performance Liquid Chromatography
MMs	Mixed Micelles
MPS	Mean Particle Size
MTT	3-(4,5-Dimethylthiazol-2-yl)-2,5-diphenyl-2H-tetrazolium bromide
P407	Poloxamer 407
P188	Poloxamer 188
PBS	Phosphate-Buffered Saline
PDI	Polydispersity Index
PEO	Poly(ethylene oxide)
PPO	Poly(propylene oxide)
TG	Thermogravimetric Analysis
XRPD	X-ray Powder Diffraction
ZP	Zeta Potential
