# Supplementary material for: Comparative Study of Fisetin-Loaded Poloxamer 407 and Poloxamer 188 Mixed Micelles as Nanocarrier Systems
Source: Molecules. 2026 May 9;31(10):1576. doi: 10.3390/molecules31101576 (PMC13210215; doi:10.3390/molecules31101576)
Supplement: Supplementary file 1 [file molecules-31-01576-s001.zip › molecules-4263313-supplementary.pdf]

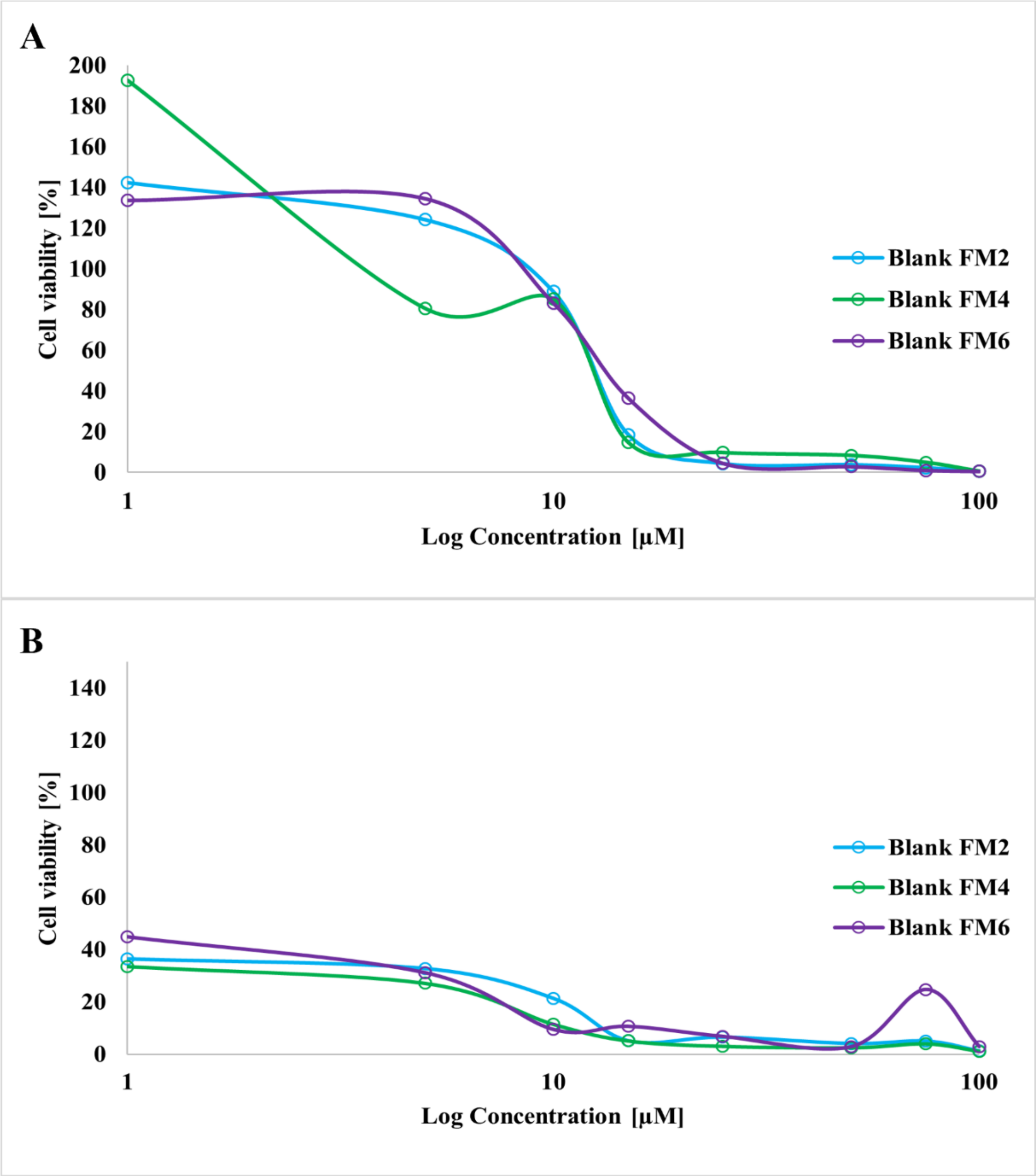

Figure S1. Cytotoxic activity of mixed micelles without FIS (blank FM2, FM4, and FM6) in (A) MICH-2 melanoma cells and (B) MRC-5 fibroblasts after 48 h incubation.
